# The role of pre-hospital blood gas analysis in trauma resuscitation

**DOI:** 10.1186/1749-7922-5-10

**Published:** 2010-04-22

**Authors:** Milla Jousi, Janne Reitala, Vesa Lund, Ari Katila, Ari Leppäniemi

**Affiliations:** 1 Department of Anaesthesiology and Intensive Care Medicine, Helsinki University Hospital, PL 340, FIN-00029 HUS, Finland; 2 Intensive Care Unit, Satakunta Central Hospital, Sairaalantie 3, FIN-28500 Pori, Finland; 3 Department of Anaesthesiology and Intensive Care Medicine, Turku University Hospital, PL 52, FIN-20521 Turku, Finland; 4 Department of Surgery, Helsinki University Hospital, PL 340, FIN-00029 HUS, Finland

## Abstract

**Background:**

To assess, whether arterial blood gas measurements during trauma patient's pre-hospital shock resuscitation yield useful information on haemodynamic response to fluid resuscitation by comparing haemodynamic and blood gas variables in patients undergoing two different fluid resuscitation regimens.

**Methods:**

In a prospective randomised study of 37 trauma patients at risk for severe hypovolaemia, arterial blood gas values were analyzed at the accident site and on admission to hospital. Patients were randomised to receive either conventional fluid therapy or 300 ml of hypertonic saline. The groups were compared for demographic, injury severity, physiological and outcome variables.

**Results:**

37 patients were included. Mean (SD) Revised Trauma Score (RTS) was 7.3427 (0.98) and Injury Severity Score (ISS) 15.1 (11.7). Seventeen (46%) patients received hypertonic fluid resuscitation and 20 (54%) received conventional fluid therapy, with no significant differences between the groups concerning demographic data or outcome. Base excess (BE) values decreased significantly more within the hypertonic saline (HS) group compared to the conventional fluid therapy group (mean BE difference -2.1 mmol/l vs. -0.5 mmol/l, p = 0.003). The pH values on admission were significantly lower within the HS group (mean 7.31 vs. 7.40, p = 0.000). Haemoglobin levels were in both groups lower on admission compared with accident site. Lactate levels on admission did not differ significantly between the groups.

**Conclusion:**

Pre-hospital use of small-volume resuscitation led to significantly greater decrease of BE and pH values. A portable blood gas analyzer was found to be a useful tool in pre-hospital monitoring for trauma resuscitation.

## Background

Hemorrhagic shock is commonly defined as a state of insufficient perfusion and oxygen supply of vital organs due to loss of blood volume and impaired cardiac preload [1,2]. In the pre-hospital setting trauma patient's shock resuscitation and its monitoring is usually based on clinical experience, assessment and a few basic parameters such as level of consciousness, blood pressure, heart rate and capillary filling time. Even if these basic clinical parameters are close to normal, shock on a cellular or organ level may be present [3-7]. There is little evidence in the literature on basic intervention strategies of fluid therapy [8-10,6]. The endpoints of shock resuscitation should be critically assessed, and resuscitation from shock considered completed only when anaerobic metabolism and tissue acidosis have been successfully reversed. The key therapeutic factor to prevent the development of multiple organ failure (MOF) is the normalisation of disturbed microvascular perfusion and oxygen supply.

Military experience and clinical and laboratory studies provide new knowledge and tools for pre-hospital and early hospital use to reverse hypovolaemia and hypoxia more effectively. Early triage, early monitoring, small-volume resuscitation with hypertonic saline, haemoglobin-based oxygen carriers, medical informatics, damage control surgery and definitive interventional radiology can be promising methods to improve the patient care [8]. Repeated measurements of arterial blood gases, lactate and haemoglobin give important information for diagnosis and follow-up. Serial haemoglobin measurements assess ongoing bleeding, and signs of metabolic acidosis indicate inadequate oxygen supply and anaerobic metabolism at cellular level, helping to evaluate the severity of shock. Pre-hospital blood gas values could as well be considered as a tool for early triage and even as criteria for trauma team activation in a hospital or a trauma centre.

This study was conducted to assess, whether measurements of blood gases before and after pre-hospital fluid resuscitation provide useful information about efficacy of resuscitation and sufficiency of perfusion and oxygenation in the tissues. The second focus of this study was to evaluate the use of small-volume resuscitation with 7.5% hypertonic saline (HS).

## Methods

In this randomised prospective preliminary study we compared two different pre-hospital fluid resuscitation strategies for severely injured patients as well as the usability and information provided by a portable blood gas analyzer. Our study included 37 adult trauma patients, who received pre-hospital care by the helicopter emergency medical system (HEMS) with an emergency physician onboard in the surroundings of Helsinki and Turku during the years 1999-2002.

The study inclusion decision was made on the accident site. The included patients were estimated to develop pre-hospitally significant hypovolaemia (>1000 ml of bleeding). Inclusion criteria were either the actual clinical state or the mechanism of injury (multiple trauma, penetrating trauma of the head, neck, chest or abdomen, fracture of pelvic ring or femur, or a suspicion of injury of large proximal vessels of the extremities). Patients, who had received more than 500 ml of crystalloids before initial assessment, were excluded. Because of the difficulty to predict the definitive diagnosis and outcome on the accident site, inclusion criteria were selected to be clear and fast to assess to find the patients in the risk of severe hypovolaemia. Not all of them were retrospectively seen as severely injured or hypovolaemic as expected, which can be interpreted from the calculated ISS and RTS-values.

The emergency physicians were using a portable clinical blood gas analyzer (i-STAT^® ^by Hewlett-Packard, nowadays a product of Abbott Laboratories) on the accident site to obtain patients' blood gas values (pH and BE) and haemoglobin level from the radial or femoral artery. According to the initial base excess (BE) value the patients were stratified into two groups (BE ≤ -3.0 mmol/l or BE > -3.0 mmol/l). In both of these groups the patients were further randomised to receive either fluid resuscitation with 300 mL of hypertonic saline (NaCl 7.5%, HS) or conventional fluid therapy (crystalloids or/and colloids). The infusion type and amount of pre-hospital conventional fluid therapy was decided by the emergency physicians, and was influenced by the levels of shock and transport time. However, the infusion protocol was essentially same in blunt and penetrating trauma patients. Data about the exact quality and quantity of the conventional fluid therapy was missing from 4 patients, all the other patients received Ringer Acetate (mean 790 ml, range 300-1300) and 7 patients received additional colloid therapy (Plasmafucin or hydroxyethylstarch 6%) (mean 380 ml, range 150-500). Hypertonic saline was administered regardless of the injury mechanism as infusion, which was targeted to end on admission to hospital. Other fluids were interrupted while HS was infused. Orion Pharma produced the hypertonic saline solution especially for this study, because at the time of the study hypertonic saline was not yet registered for pharmacological use in Finland.

Patient's blood pressure and heart rate were measured every 10 minutes during transport to the hospital. Blood gas values were measured again on admission to hospital with a subsequent lactate level measurement.

Revised Trauma Score (RTS) and Injury Severity Score (ISS) were calculated retrospectively based on the patients' pre-hospital notes and the hospital records [11-14].

Data are presented as mean (standard deviation) for continuous and as proportions for discrete values. Continuous variables with normal distribution were analysed with the Student's t test, and non-normally distributed variables with the Mann-Whitney U-test. Proportions were compared with Fisher's exact test. SPSS^® ^statistics software was used for calculations. The statistical significance level is agreed at p < 0.05.

## Results

Seventeen patients (46%) received hypertonic fluid resuscitation and 20 (54%) conventional fluid therapy. There was no statistically significant difference between the two groups concerning age, sex, mechanism of injury, incidence of brain injury, RTS or ISS (Table 1). The mean (SD) age of the patients was 44 (21 - range 16-87) years, 29 (78%) of them were male. Four patients (11%) had a penetrating injury (2 gunshot wounds, 1 stabbing, 1 explosion), and 33 (89%) had blunt injuries (22 traffic accidents, 7 falls, 3 compression injuries and one patient injured by a heavy falling object). The mean RTS was 7.3427 (0.98) (range 4.09 - 7.84), and mean ISS was 15.1 (11.7) range 1-41). Eighteen patients (49%) were treated at the Turku University hospital and 19 (51%) at the Helsinki University Hospital. Nine patients (24%) had a brain injury. The overall mortality rate was 3 (8%) patients. The outcome variables did not differ between the two treatment groups (Table 2).

**Table 1:** Patient characteristics

	Overall	Hypertonic Saline (HS) group	Conventional fluid therapy group	p-value
Number of patients	37	17 (46%)	20 (54%)	

Mean patient age in years (SD)	44 (21)	37 (18)	50 (22)	0,074

Number of male patients (percentage)	29 (78%)	12 (71%)	17 (85%)	0,428

Number of female patients (percentage)	8 (22%)	5 (29%)	3 (15%)	

Number of patients with blunt trauma (percentage)	33 (89%)	15 (88%)	18 (90%)	1,000

Number of patients with penetrating trauma (percentage)	4 (11%)	2 (12%)	2 (10%)	

Number of patients with associated brain injury (percentage)	9 (24%)	5 (29%)	4 (20%)	0,703

Mean Injury Severity Score ISS (SD)	15,1 (11,7)	13,4 (9,5)	16,5 (13,3)	0,614

Mean Revised Trauma Score RTS (SD)	7,343 (0,977)	6,949 (1,302)	7,680 (0,369)	0,084

Mean Glasgow Coma Score GCS (SD)	13,0 (3,2)	12,6 (3,4)	13,3 (3,1)	0,374

Time interval in minutes from trauma to BE-measurement on accident site (SD)	47 (22)	48 (21)	45 (23)	0,372

Time interval in minutes from BE-measurement on accident site to hospital admission (SD)	53 (27)	60 (29)	47 (24)	0,106

**Table 2:** Outcome

	Overall	Hypertonic Saline (HS) group	Conventional fluid therapy group	p-value
Mortality (percentage)	3 (8%)	1 (6%)	2 (10%)	1.000

Transfused red blood cell units (SD)	5.4 (8.5)	4.4 (8.7)	6.2 (8.3)	0.416

Duration of intensive care in days (SD)	5 (8)	5 (7)	6 (9)	0.670

Duration of hospital care in days (SD)	25 (43)	15 (12)	34 (57)	0.891

In both groups, the systolic blood pressure and heart rate values increased from the accident site to the time of the hospital admission, but there was no difference between the two fluid strategy groups (Table 3). In contrast, the BE levels decreased more within the HS group (mean BE difference -2.1), than in the conventional fluid therapy group (mean BE difference -0.5) (p = 0.003). The pH value on admission was significantly lower within the HS group (mean 7.31 vs. 7.40, p = 0.000). The haemoglobin levels were lower in both groups on admission compared to the accident site, and more within the HS group (mean -22 vs. -11, p = 0.016). Lactate levels on admission did not differ significantly between the groups (Table 3).

**Table 3:** Results

	Overall	Hypertonic Saline (HS) group	Conventional fluid therapy group	p-value
Mean of Systolic Blood Pressure values on accident site in mmHg (SD)	122 (29)	118 (32)	125 (26)	0.293

Mean of Systolic Blood Pressure values on admission to hospital in mmHg (SD)	141 (26)	141 (26)	141 (28)	0.945

Mean of change in Systolic Blood Pressure values in mmHg between accident site and admission to hospital (SD)	21 (30)	27 (35)	17 (26)	0.652

Mean of Heart rate values (beats per minute) on accident site (SD)	86 (20)	86 (20)	86 (22)	0.976

Mean of Heart rate values on admission to hospital (SD)	93 (25)	99 (23)	88 (25)	0.241

Mean of change in Heart rate values between accident site and admission to hospital (SD)	7 (17)	12 (20)	3 (14)	0.248

Mean of Base Excess values (BE) (mmol/L) on accident site (SD)	-2.6 (4.0)	-2.8 (4.1)	-2.4 (4.1)	0.866

Mean of Base Excess values (BE) (mmol/L) on admission to hospital (SD)	-3.3 (3.4)	-5.0 (2.8)	-1.9 (3.3)	0.008 *

Mean of differences in Base Excess values between accident site and admission to hospital (SD)	-0.6 (2.8)	-2.1 (2.6)	-0.5 (2.4)	0.003 *

Mean of pH values on accident site (SD)	7.38 (0.09)	7.35 (0.11)	7.41 (0.07)	0.205

Mean of pH values on admission to hospital (SD)	7.36 (0.08)	7.31 (0.07)	7.40 (0.06)	0.000 *

Mean of differences in pH values between accident site and admission to hospital (SD)	-0.03 (0.09)	-0.04 (0.12)	-0.01 (0.05)	0.196

Mean of Haemoglobin values (Hb) (g/L) on accident site (SD)	135 (17)	135 (17)	135 (17)	0.963

Mean of Haemoglobin values (Hb) (g/L) on admission to hospital (SD)	119 (19)	114 (20)	124 (17)	0.074

Mean of differences in Haemoglobin values between accident site and admission to hospital (SD)	-16 (14)	-22 (14)	-11 (12)	0.016 *

Mean of patient Lactate levels (mmol/L) on admission to hospital (SD)	2.34 (1.37)	2.21 (1.26)	2.46 (1.49)	0.871

## Discussion

There are numerous studies with different focuses on pre-hospital blood gas analysis in patients undergoing out of hospital cardiopulmonary resuscitation [15-18] or during emergency transport [19]. In addition, there are several studies about predictive value of lactate, pH and BE in severely injured trauma patients [20-22], but the measurements are all made after admission to a hospital. In an Austrian prospective study about small-volume resuscitation, repeated measurements of venous blood electrolytes, haemoglobin and white cell count were performed, but arterial blood-gas values were not measured [23]. This study attempts to obtain more information about pre-hospital arterial blood-gas analysis and shock resuscitation of severely injured trauma patients.

Blood lactate levels have been shown to correlate with injury severity as well as the overall prognosis of the severely injured patient [20]. Kaplan et al. were able to show among 282 patients with a major vascular injury, that initial emergency department acid-base variables (pH, base deficit, lactate, anion gap, apparent strong ion difference and strong ion gap) were able to discriminate survivors from non-survivors [21]. Sindert et al. published recently a large study with 489 trauma patients, where they were testing the diagnostic utility of Base Deficit (BD) measurements at triage and four hours later, in distinguishing minor from major injury [22]. They wanted to test, if infusion of chloride-rich solution, such as normal saline (NS), confuses the results. Even infusion of more than 2000 ml of normal saline didn't confound the prognostic value of BD.

In this study, there were clear differences in BE and pH values between the two different fluid strategy groups. The reason for this difference remains unclear. Considering BE and pH values as markers of adequate tissue oxygenation, conventional fluid therapy appears to be more effective than small volume resuscitation in compensating the hypovolaemia. Because 300 ml of hypertonic saline (NaCl 7.5%) contains 385 mmol of chloride ions (1283 mmol/l), it could cause hyperchloraemic acidosis. Chloride levels were not measured in this study. There was no statistically significant difference between the lactate levels, which would support some other cause for the acidosis than lactataemia and compromised tissue oxygenation. The greater decrease of the haemoglobin level within the HS-group is presumably explained by a larger intravascular volume effect of the HS and haemodilution. There is evidence, that infusion of hypertonic saline dextran causes metabolic acidosis. Kreimeier and Messmer in their review article suggest, that acidosis after bolus infusion of hypertonic saline would be due to improvement of nutritional blood flow and a wash-out of acidic substances and metabolites, rather than only hyperchloraemia [24].

There has been an extensive interest in hypertonic saline during the past few decades because of its ease of transport, logistical feasibility for military use, speed of administration and rapid correction of haemodynamics [25]. In fluid resuscitation the basic mechanism of action of hypertonic saline is rapid osmotic mobilisation of water from intercellular spaces, endothelial cells and red blood cells into intravascular space. Because cells become oedematous during shock, hypertonic saline has been shown to normalize cell volume rather than reduce it below normal. Infusion of hypertonic saline dilates arterioles and reduces peripheral and pulmonary vascular resistance by directly relaxing smooth muscle and decreasing blood viscosity. Heart rate and cardiac contractility are both increased, and all that synergistically increases cardiac output and oxygen delivery to the tissues [24-26]. Combining a colloid component to hypertonic saline, nowadays most frequently 6% dextran 70, results in a significantly higher cardiac output and more sustained plasma volume expansion. In recent animal and in vitro studies hypertonicity has been found to affect immune responses of trauma, shock and reperfusion by suppressing several neutrophil functions and up-regulating T-lymphocyte functions. Hypertonic saline has been shown to cause key alterations in interactions of polymorphonuclear neutrophils and endothelial cells, which under shock conditions (mediated by proteases and free oxygen radicals) are partly responsible for development of systemic inflammatory response syndrome (SIRS). Also, hypertonic saline has been shown to decrease microvascular permeability [25,27]. Hypertonic saline could be considered both as a resuscitation fluid for restoring intravascular volume as well as an immunomodulator to prevent later complications, such as multiple organ failure (MOF).

Even if there is evidence of hypertonic resuscitation concerning safety [23,28,29] and effectiveness in restoring macrovascular haemodynamics, large human clinical trials have not yet been able to demonstrate consistently benefit in terms of morbidity or mortality [30-32]. The results about long-term benefit for patients with traumatic brain injury are contradictory [33-35]. On the other hand patients, who were hypotensive and required surgery because of penetrating injuries to the torso, had improved survival if they received hypertonic saline instead of conventional fluid therapy [36]. Mortality might though not represent the optimal end point for studies for small-volume resuscitation. Rather, measures of organ dysfunction might show its real benefits [24,37].

We found out some weaknesses in our study setting. One is, that despite of the tight inclusion criteria, which were supposed to find the hypovolaemic patients, many of them were though not severely injured, as can be seen with ISS and RTS-values. Another confusing factor is the variety of pre-hospital circumstances. The two emergency helicopters are covering a very large geographical area with varying quality of baseline emergency services. Patients from remote locations are though transported primarily to Level 1 Trauma Centre with an ambulance and an emergency physician, which causes sometimes relatively long pre-hospital times.

Studies with more patients are needed to show the real reason and significance of the differences in BE and pH values between the patients receiving different types of fluid resuscitation. Electrolyte measurements with blood-gas values are needed to determine more precisely the type of acidosis. Correlation between injury severity and initial pre-hospital BE and pH could be examined in order to consider blood-gas values as a tool for triage. Taking arterial blood samples and using a portable clinical blood gas analyzer at the accident site requires additional time and efforts from the emergency physician, and its usefulness should be judged in view of the overall time and resource utilization. This study, however, shows that arterial blood gas analyses in the field are feasible and could be used in the future for better en-route management and triage for severely injured patients.

## Conclusions

Pre-hospital arterial blood gas measurements during trauma patient's fluid resuscitation by emergency physician based helicopter emergency medical system (HEMS) provided useful information about patients' acid-base values. Comparing the values after either conventional fluid therapy or small-volume resuscitation with hypertonic saline demonstrated, that the use of small-volume resuscitation lead to significantly greater decrease in the BE and pH values. The reason for this remains unclear. A portable clinical blood gas analyzer (i-STAT^® ^by Hewlett-Packard) was found to be a usable tool for pre-hospital monitoring of trauma resuscitation.

## Competing interests

The authors declare that they have no competing interests.

## Authors' contributions

JR, VL, AK and AL have been participating in the study design. JR, VL and AK have been participating in the data collecting on field. MJ performed the data collection from the patient files, performed the statistical analysis and completed the manuscript with the support of AL. All authors have read and approved the final manuscript.
